# Save the Leg: Utilization of Distal Perfusion Catheter With Impella CP® May Prevent Morbidity of Limb

**DOI:** 10.7759/cureus.29916

**Published:** 2022-10-04

**Authors:** Lydia McDermott, Gary Cook, Joshua Park, Qiong Yang, Hitoshi Hirose

**Affiliations:** 1 Surgery, Virtua Health, Camden, USA

**Keywords:** isolated limb perfusion, imeplla, shcok, ecmo, leg ischemia

## Abstract

Leg ischemia is a potential complication of percutaneous left ventricular assist device (Impella CP®) placement. To avoid leg ischemia in at-risk patients, a distal perfusion catheter (DPC) should be placed. In utilizing a passive distal perfusion system from the contralateral femoral artery, we optimized blood flow to the distal limb mitigating leg ischemia. A 65-year-old female with dilated cardiomyopathy complicated by hemodynamic instability was placed on an Impella CP via the right femoral artery. A DPC was placed to the right distal femoral artery and connected to the wire re-access port of the Impella CP. Despite this, the leg became ischemic shortly after admission to the ICU. A contralateral femoral arterial line was placed in standard fashion, and it was connected to the DPC while the wire re-access port was capped. Shortly after placement of the new DPC system, the right lower extremity distal pulses returned, and distal leg ischemia was resolved. Another patient, a 67-year-old male with acute myocardial infarction, was placed on an Impella CP via the left femoral artery for cardiogenic shock. His hemodynamics continued to deteriorate, requiring initiation of veno-arterial extracorporeal membrane oxygenation (VA ECMO) via the right femoral artery and vein with associated DPC placement. Shortly after the initiation of VA ECMO, the Impella CP-related extremity (left leg) became ischemic. A left femoral DPC was placed and connected to the side port of the right femoral arterial cannula. After initiation of the additional DPC system, the left leg ischemia resolved. Distal leg ischemia with Impella CP is not a rare event. Utilization of a DPC to Impella CP may decrease the morbidity of limb malperfusion.

## Introduction

Leg ischemia is a possible complication of percutaneous left ventricular assist device (Impella CP®, Abiomed, Danvers, Massachusetts, United States) placement. To avoid leg ischemia in patients with small femoral arteries, a distal perfusion catheter (DPC) should be placed. The DPC can be connected to the wire re-access port of the Impella CP as an inflow to the DPC. However, the re-access port was not designed to bypass arterial blood, but rather was created for wire passage and as a result, may impair arterial flow. We introduced a passive distal perfusion system from the contralateral femoral artery to optimize blood flow in the Impella CP-related limb. In addition, leg ischemia may occur in either limb for patients on both an Impella and on extracorporeal membrane oxygenation (ECMO). To avoid distal limb ischemia in this setting, bilateral DPC placement via a side-port on the arterial ECMO cannula may be necessary.

This article was previously presented as a meeting abstract at the 67th annual conference American Society for Artificial Organs, June 8-11, 2022 [1].

## Technical report

Case 1 (Impella CP and DPC)

**Figure 1:**
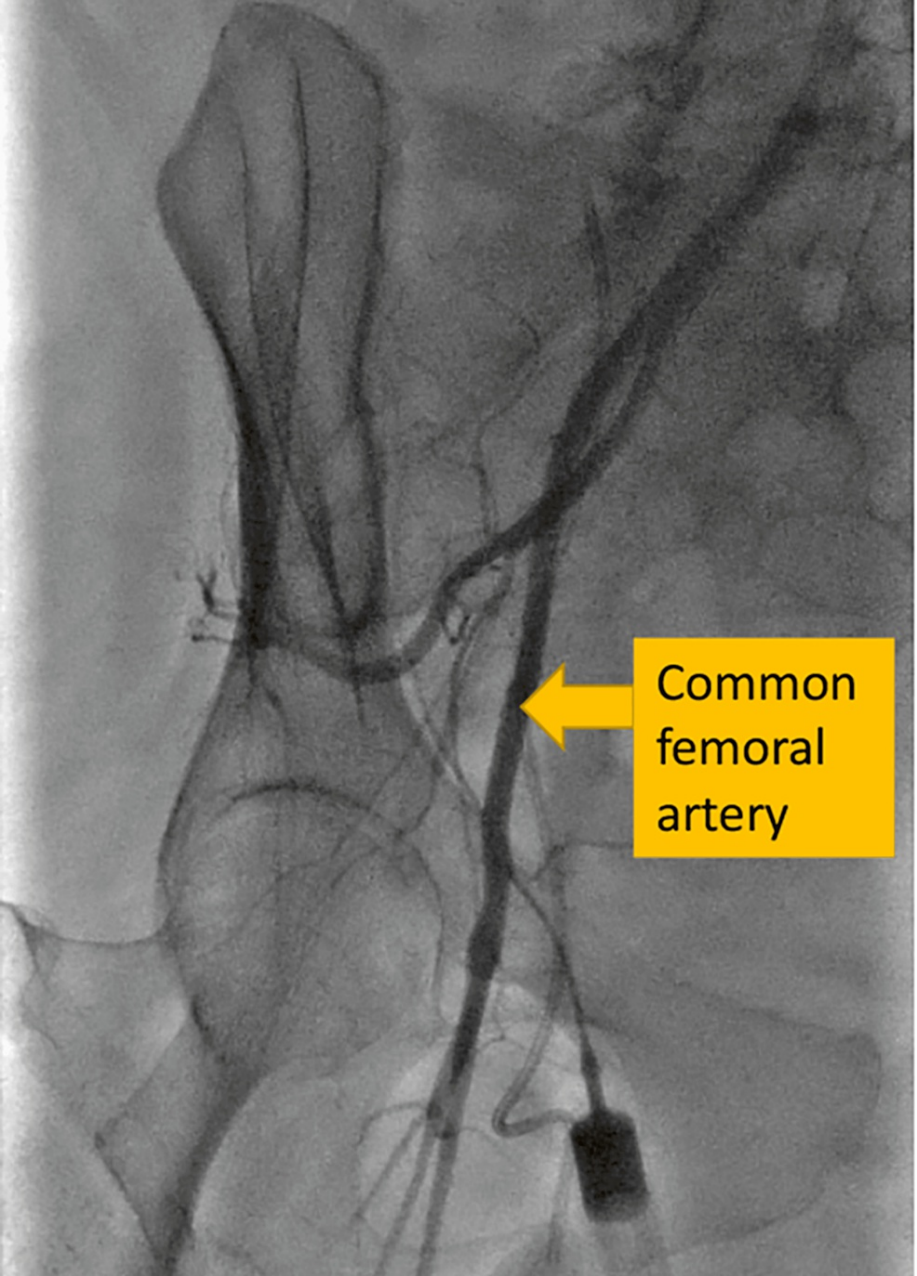
Femoral angiography Femoral angiography shows a small common femoral artery; placement of Impella CP to such small arteries most likely causes distal limb ischemia.

A 65-year-old female with dilated cardiomyopathy developed refractory ventricular tachycardia and cardiogenic shock requiring high doses of vasopressors. She was taken to the catheterization laboratory where she was noted to have elevated left ventricular end-diastolic pressure (38 mmHg) and a poor cardiac index despite normal coronary anatomy. An Impella CP was placed via the right femoral artery to stabilize her hemodynamics. A DPC was placed to the right distal femoral artery using a 5 Fr sheath under ultrasound guidance, although the femoral artery appeared small by angiogram (Figure 1).

**Figure 2:**
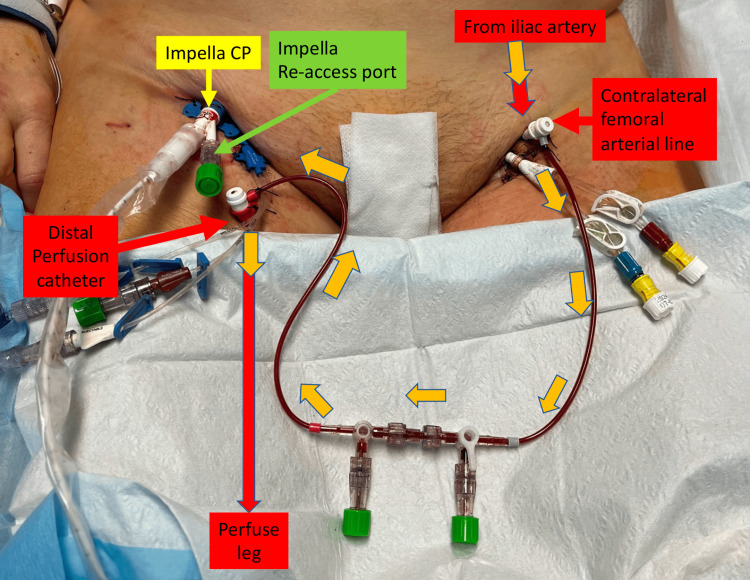
DPC for Impella CP®: Distal perfusion catheter placement on leg affected by Impella side DPC: distal perfusion catheter placement

**Figure 3:**
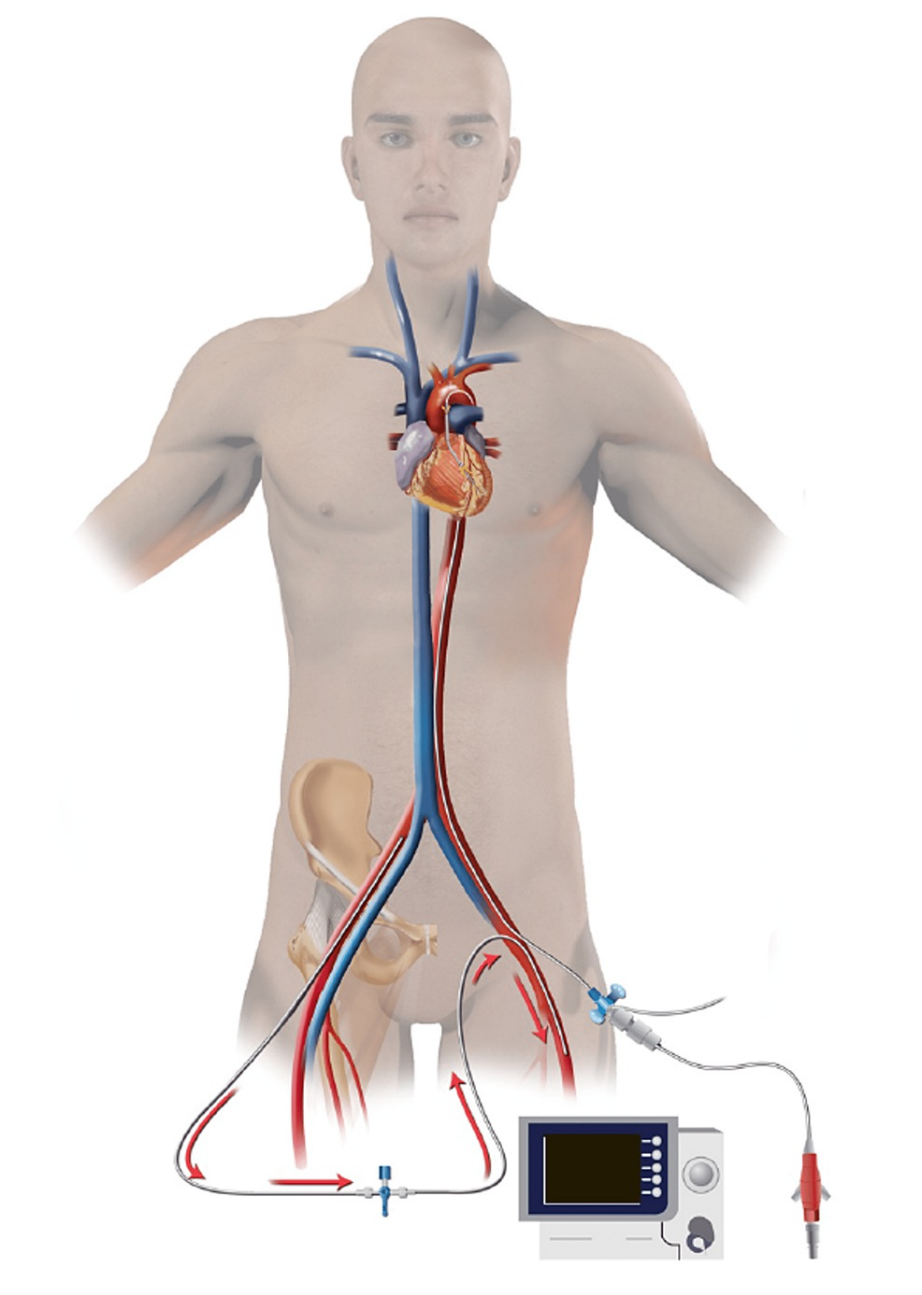
Illustration of distal perfusion catheter placement for patients with a femoral Impella Image: Author (HH) has copyright

The DPC was connected to the wire re-access port of the Impella CP. The leg was perfused by the DPC via the re-access port in the catheterization laboratory; however, despite initial good flow and appropriate anticoagulation, the leg became ischemic shortly after admission to the ICU. There appeared to be malperfusion of the leg (distal pulses were not detected and the right leg became cold), which was further confirmed by a drop in the tissue oxygen saturations and loss of distal pulses. The DPC was flushed and patency was confirmed. Upon flushing of the re-access port, there was only sluggish flow likely not enough to perfuse the entire leg. An additional contralateral femoral arterial line was placed using a 5 Fr sheath and connected to the DPC while the Impella re-access port was capped (Figure 2, Figure 3).

Shortly after placement of the new DPC system, the distal right pulse returned to baseline and the right thigh and calf oxygenations improved to the level found on the contralateral side. Limb ischemia did not reoccur while on Impella CP support with this DPC configuration in place.

Case 2 (Impella CP, ECMO, and DPC)

**Figure 4:**
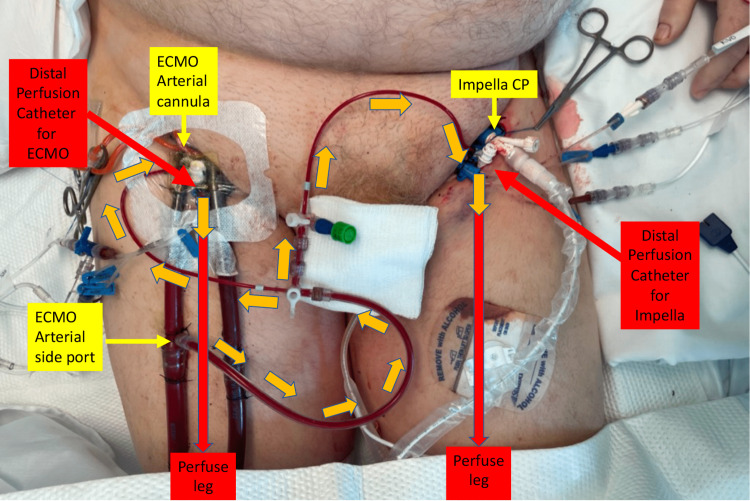
DPC for ECMO with Impella CP®: Bilateral distal perfusion catheter placement to the veno-arterial ECMO and Impella limbs DPC: distal perfusion catheter; ECMO: extracorporeal membrane oxygenation

**Figure 5:**
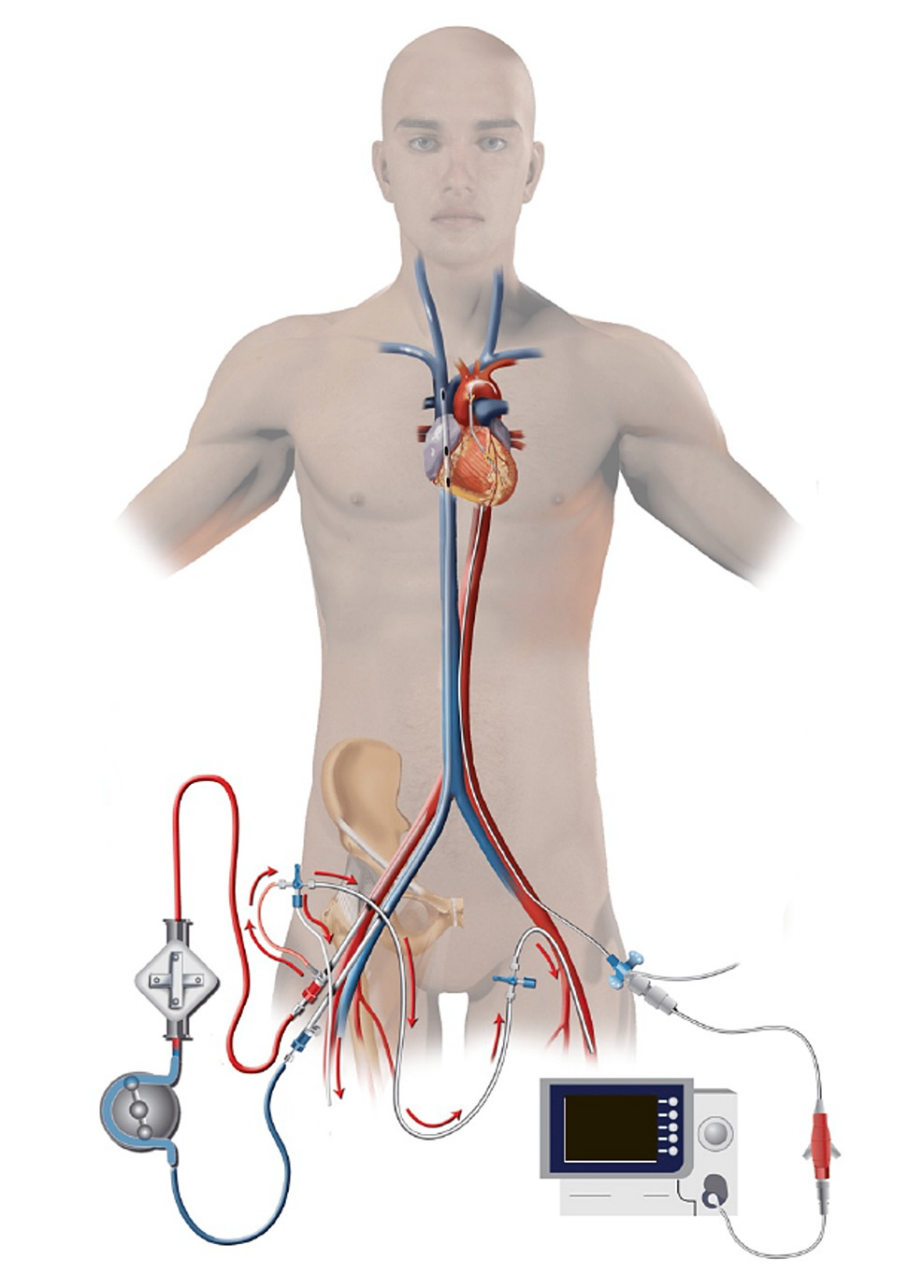
Illustration of distal perfusion catheter placement for the patient with concomitant veno-arterial ECMO and Impella use Image: Author (HH) has copyright

A 67-year-old male was admitted to the hospital with acute anterior ST-elevation myocardial infarction. He was intubated in the emergency room due to acute pulmonary edema and sent to the catheterization laboratory. Due to ongoing hemodynamic instability, an Impella CP was placed through the left femoral artery. Coronary angiography revealed a proximal left anterior descending artery (LAD) occlusion, which was successfully revascularized with coronary stents. Despite this intervention, his hemodynamics continued to deteriorate requiring initiation of veno-arterial extracorporeal membrane oxygenation (VA ECMO) via the right femoral artery and vein with subsequent DPC placement. Shortly after the initiation of VA ECMO, the Impella CP-related limb became ischemic with the development of a cold leg and loss of distal pulses. An additional DPC was placed in the left distal femoral artery under ultrasound guidance and connected to the VA ECMO DPC system (Figure 4, Figure 5).

The left distal pulse was restored and there were no further episodes of leg ischemia during VA ECMO/Impella CP support. The patient remained on VA ECMO/Impella CP support for five days until he developed a fatal intracranial hemorrhage.

## Discussion

Impella CP has been used for cardiogenic shock patients to improve cardiac output and promote cardiac recovery. Impella CP is a catheter-based, mechanical circulatory assist device (MCS) that is placed percutaneously via the femoral artery. The catheter tip lies in the left ventricle and suctions blood from the left ventricle and disperses it into the ascending aorta. Impella CP support provides left ventricular unloading, increases systemic perfusion, and increases coronary blood flow [2]. Among the variety of Impella catheters, Impella CP is most often used in the catheterization laboratory since it allows for percutaneous insertion and provides a maximum of 3.7 L/min of flow, which is appropriate in most cases of cardiogenic shock. The catheter size is 9 Fr and can be often placed without distal leg compromise; however, patients with a small femoral artery can suffer from distal leg ischemia. Furthermore, patients in cardiogenic shock often require high doses of vasopressors, which constrict arteries, further compromising distal leg perfusion [3]. The reported incidence of distal leg ischemia with Impella CP is 4-17% and is more common in females, due to small caliber femoral arteries, or in older patients, likely as a result of the presence of baseline peripheral vascular disease [4]. Prompt diagnosis of leg ischemia and efficient placement of the DPC is important to preserve at-risk limbs [5,6]. Since this vascular complication is so significant, routine assessment of the femoral artery before Impella CP placement and early DPC insertion in high-risk patients may be necessary. Furthermore, the risk of limb ischemia becomes increasingly important in patients requiring prolonged Impella CP support compared to those who undergo short-term Impella CP support for the purpose of facilitating high-risk interventions. 

Concomitant use of Impella CP with VA ECMO (known as ECpella™) has recently gained attention as a way to vent the left ventricle. VA ECMO provides quick, complete circulatory and respiratory support, while Impella CP provides left ventricular support alone. VA ECMO is commonly placed peripherally via the femoral artery and vein. Leg ischemia in VA ECMO has been repeatedly reported, though the placement of a DPC to the affected limb could mitigate related leg ischemia [7,8]. However, peripherally cannulated VA ECMO functions by providing retrograde blood flow in the aorta and can increase left ventricular wall stress, causing distention and blood stasis in the left ventricle. This has been described to adversely affect the recovery of cardiac function. Appropriate use of Impella CP in combination with VA ECMO (ECpella) could potentially unload the left ventricle, facilitate cardiac recovery, and improve patient outcomes [9], though, ischemia could occur in the Impella CP-related limb depending on the femoral artery size and possible vasospasm due to the use of vasopressors. Furthermore, the lack of pulsatile flow caused by continuous VA ECMO flow could accelerate leg ischemia in a compromised limb. Once leg ischemia is detected, the patient should be treated immediately to potentially prevent ischemic complications such as rhabdomyolysis, acute renal failure, or even limb loss. Bilateral DPC placement during ECpella cannulations was advocated by Kizner et al. [10] to minimize these effects. Alternatively, if the patient’s cardiac/respiratory function is improved, VA ECMO could be removed and the patient could then be managed by an Impella CP alone. In that case, DPC inflow for perfusion of the Impella-affected limb should be established from the femoral artery on the opposite side where ECMO arterial cannula was placed. Placement of DPC while surgical VA ECMO decannulation would be easy since the artery is exposed during the procedure. An additional arterial line may need to be obtained if a percutaneous closure device is used for decannulation. 

## Conclusions

Distal leg ischemia can occur in patients with small vasculature and vasospasm due to vasopressor use in the setting of femoral Impella CP use. DPC placement with appropriate inflow can prevent progression of distal leg ischemia even with concomitant VA ECMO use.
